# Coupling the Paternò-Büchi (PB) Reaction With Mass Spectrometry to Study Unsaturated Fatty Acids in Mouse Model of Multiple Sclerosis

**DOI:** 10.3389/fchem.2019.00807

**Published:** 2019-11-26

**Authors:** Leelyn Chong, Ran Tian, Riyi Shi, Zheng Ouyang, Yu Xia

**Affiliations:** ^1^State Key Laboratory of Crop Stress Adaptation and Improvement, State Key Laboratory of Cotton Biology, School of Life Sciences, Henan University, Kaifeng, China; ^2^Department of Chemistry, Purdue University, West Lafayette, IN, United States; ^3^Weldon School of Biomedical Engineering, Purdue University, West Lafayette, IN, United States; ^4^Department of Basic Medical Sciences, College of Veterinary Medicine, Purdue University, West Lafayette, IN, United States; ^5^State Key Laboratory of Precision Measurement Technology and Instruments, Department of Precision Instrument, Tsinghua University, Beijing, China; ^6^Department of Chemistry, Tsinghua University, Beijing, China

**Keywords:** fatty acids, isomers, spinal cord, hydralazine, experimental autoimmune encephalomyelitis (EAE), multiple sclerosis

## Abstract

Lipid dysregulation has been implicated in multiple sclerosis due to its involvement during and after inflammation. In this study, we have profiled fatty acids (FAs) in the mouse model of multiple sclerosis with new capabilities of assigning carbon-carbon double bond (C=C) location(s) and quantifying C=C location isomers. These new capabilities are enabled by pairing the solution phase Paternò-Büchi (PB) reaction that modifies C=C bonds in FAs, with tandem mass spectrometry (MS/MS), termed as PB-MS/MS. A series of unsaturated FAs and C=C location isomers have been identified, including FA17:1 (Δ10), FA18:1 (Δ9 and Δ11), FA18:2 (Δ9 and Δ12), and FA 20:4 (Δ5, Δ8, Δ11, Δ14). Notable differences in saturated and unsaturated FAs between normal and experimental autoimmune encephalomyelitis (EAE) mice spinal cords have been detected. Furthermore, the effects of hydralazine, a scavenger of acrolein, on profile changes of FAs in mice were studied. Increased Δ11-to-Δ9 isomer ratios for FA 18:1 were noted in the diseased samples as compared to the control. The present work provides a facile and robust analytical method for the quantitation of unsaturated FAs as well as identification of FA C=C location isomers, which will facilitate discovering prospective lipid markers in multiple sclerosis.

## Introduction

Lipids are one of the most essential biomolecules that living organisms cannot survive without. Lipids are structurally diverse, containing a variety of isobars and isomers, which makes lipid identification and quantitation challenging by conventional analysis methods (Biochemistry of lipids, 1953; Wenk, 2010). The unsaturated lipids have been known to influence the development and progression of numerous health disorders (Wymann and Schneiter, 2008). Unfortunately, the lack of a fast and robust method for determining the identity of unsaturated lipids, especially with accurate information of carbon-carbon double bonds (C=C) locations, hinders the studies of the evolving area of structural and functional lipidomics (Porta Siegel et al., 2019). Recently, our groups and others have demonstrated that by coupling C=C selective derivatization via the photochemical Paternò-Büchi (PB) reaction with subsequent tandem mass spectrometry (MS/MS) analysis, the locations of C=C bonds in unsaturated fatty acyls can be confidently determined in a sensitive and high-throughput fashion (Ma and Xia, 2014; Ma et al., 2016a,b; Murphy et al., 2017; Ren et al., 2017; Franklin et al., 2019; Zhang et al., 2019). Due to the unique capabilities of this method, one can now choose to perform direct analysis of the lipid mixtures extracted from biological samples; especially if the individual is looking for a rapid disease diagnosis (Ma et al., 2016a; Zou et al., 2019).

In a previous study, it was determined that the lipid C=C isomeric ratios could potentially serve as biomarkers for disease diagnosis. In this investigation, we explored the potential of this method for study of multiple sclerosis. Multiple sclerosis is a chronic inflammatory and demyelinating disease of the central nervous system (Dendrou et al., 2015; Pappalardo and Hafler, 2019). Multiple factors have been indicated for disease emergence and re-emergence, including autoimmune etiology, environmental factors, as well as specific genetic predispositions (Abbasalizad Farhangi et al., 2019; Agresti et al., 2019; Ahumada-Pascual et al., 2019; Kotelnikova et al., 2019). Additionally, the neurological damage in multiple sclerosis is insinuated to be of a neurodegenerative nature (Ortiz et al., 2013; Binyamin et al., 2015). Myelin is a lipid-rich substance that insulates nerve fibers and facilitates electrical communication between neurons (O'Muircheartaigh et al., 2019). Lipid molecules have been indicated to act as target molecules of myelin damage and mediators of inflammation in multiple sclerosis (Miller and Karpus, 2007; Brennan et al., 2011; Multiple sclerosis, 2018). Fatty acids (FAs) serve as the major constituents of complex lipids such as phospholipids and glycolipids in biological membranes (van Meer et al., 2008; Wymann and Schneiter, 2008). Homeostasis of FAs in a lipidome is vital to the survival of living organisms as membrane integrity, fluidity, permeability, along with the activities of membrane bound enzymes in cells are influenced by FAs (van Meer et al., 2008; Phillips et al., 2009). Moreover, FAs have been indicated to play a role in preventing many disorders as they have nutritional relevance in the mammalian diet (Adamo, 2014; Dendrou et al., 2015).

**Scheme 1:**
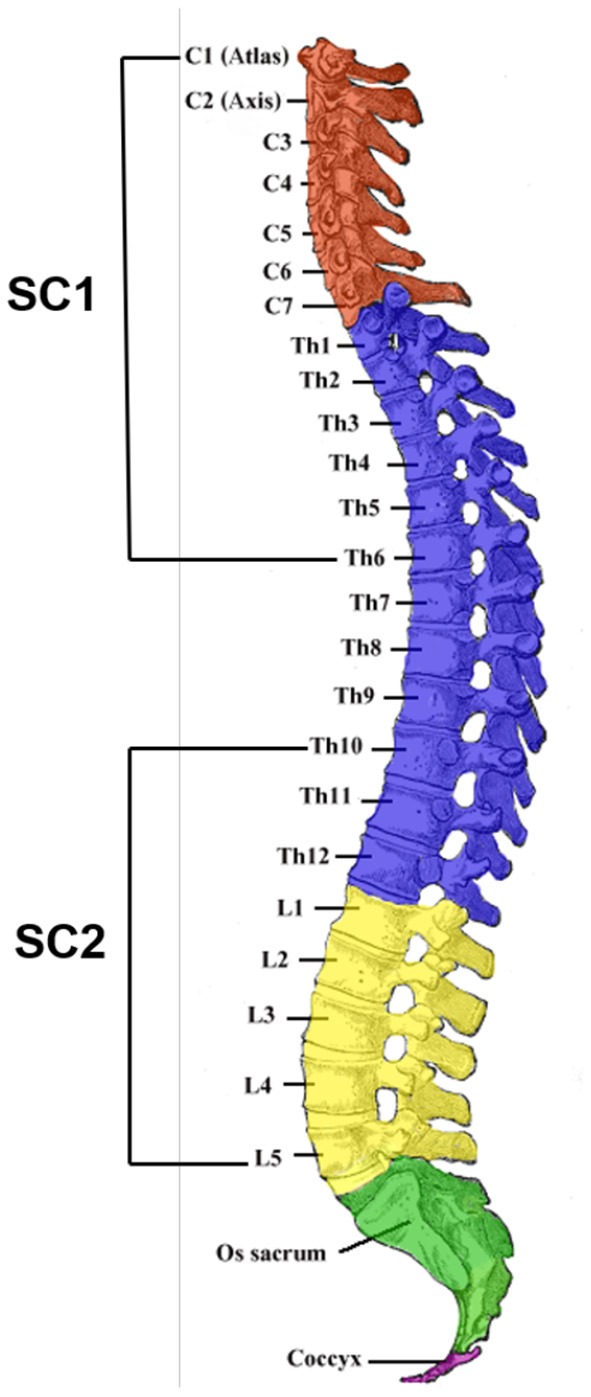
The four divisions [cervical (C), thoracic (Th), lumbar (L), and sacral (S)] of the vertebral column. Surgical removal of SC1 and SC2 portions of the mouse spinal cord was performed and C1–C7 and Th1–Th6 segments were obtained together as the SC1 portion whereas Th10–Th12 and L1–L5 segments were acquired subsequently as the SC2 portion. The SC1 portion of the spinal cord is responsible for upper bodily functions such as breathing and heart movement whereas the SC2 portion has major functions in controlling the behaviors and movement of the lower body (Skup et al., 2007). (*Image of the spinal column was extracted from Lumen Learning and modified for the purpose of illustrating the attained portions for this research*).

To date, the experimental autoimmune encephalomyelitis (EAE) animal model best mimics the clinical and pathological hallmarks of multiple sclerosis and can offer the essential predictive guide for clinical therapeutic application (Miller and Karpus, 2007; Quinn and Axtell, 2018). Significantly elevated concentrations of acrolein, an α, α-unsaturated aldehyde and a reactive product of lipid peroxidation, has been found after rat spinal cord contusion injury. Given that hydralazine is a proven scavenger of acrolein and it offers *in vivo* neuroprotection (Due et al., 2014; Tully et al., 2014; Chen et al., 2016; Butler et al., 2017; Lin et al., 2018), it is interesting to monitor the change of FA profiles of the spinal cords before and after the treatment of hydralazine to EAE mice. Herein, we have developed a shotgun lipid analysis approach, with online photochemical reaction incorporated, to investigate the significance of FAs and their isomers in two various segments of the spinal cord (SC1 and SC2) in the EAE animal model (Scheme 1).

## Materials and Methods

### Preparation of Animals for Experiments

Eight weeks old of C57BL/6 female mice were purchased from Harlan Laboratories (Indianapolis, IN, USA) and were housed in the lab animal housing facilities. These studies were performed in compliance with the Purdue Animal Care and Use Committee protocol guidelines at Purdue University, West Lafayette, Indiana. The animals were acclimated for at least 1 week prior to performing any operations.

### Induction of Experimental Autoimmune Encephalomyelitis in Mice

Ten weeks or older of female C57BL/6 mice were subcutaneously immunized with an emulsion containing 0.1 mL of 1 mg/mL MOG_35−55_/CFA (EK-0115, Hooke Laborites, Lawrence, MA). The inoculum (0.2 mL/mouse total) was subcutaneously injected over both the rostral and caudal ends of the spinal cord. This mixture mimicked endogenous proteins and created an immune response to the myelin in the central nervous system. After 2 h, 0.1 mL of 400 ng deconjugated pertussis toxin (Hooke Laboratories, Lawrence, MA) was administered intraperitoneally and then the second dose was given 24 h later. The pertussis toxin was used to create a permeable blood brain barrier and accelerated the onset of symptoms.

### Behavioral Assessment Using the EAE Scoring System

To observe for the appearance of neurological systems, the mice's behavioral assessment was performed using a 5-point scoring system (Kalyvas and David, 2004). Animals were placed on a metal grate and their walking ability was observed. The scale is as follows: 0—no deficit; 1—limp tail only; 2—hind limb paresis without frank leg dragging; 3—partial hind limb weakness with one or both legs dragging; 4—complete hind limb paralysis; 5—moribund, paralysis in hind limbs and forelimbs. The animals were monitored every other day during the first week and then daily until the end of the study.

### Preparation of Hydralazine Treatment

Hydralazine hydrochloride salt (Sigma Aldrich, St. Louis, MO) was dissolved in phosphate buffered saline and then sterilized through a filter. Hydralazine solution (1 mg/kg) was stored at 4°C and intraperitoneally administered on the same day of induction and then daily until the end of the study. Control animals were also given the same treatment of saline.

### Animal Sacrifice and Specimen Collection

The animals were anesthetized by injecting intraperitoneally with a mixture of ketamine (90 mg/kg) and xylazine (10 mg/kg). After completely anesthetized, animals were perfused with oxygenated Kreb's solution including 124 mM NaCl, 2 mM KCl, 1.24 mM KH_2_PO_4_, 26 mM NaHCO_3_, 10 mM ascorbic acid, 1.3 mM MgSO_4_, 1.2 mM CaCl_2_, and 10 mM glucose. The whole vertebral column was rapidly removed and the laminectomy was performed. The spinal cord was removed and separated into upper and lower sections around the T10 level.

### Analysis and Extraction of Fatty Acids

Synthetic standards of FAs were purchased commercially (Avanti Polar Lipids, Inc. and Nuk Prep, Inc.). FAs in the spinal cord tissues from both normal and EAE mice in various disease stages were extracted by methanol and iso-octane (Bligh and Dyer, 1959). The fatty acids from mice treated with hydralazine were also obtained in the similar approach for lipid analysis. Both the standards and extracts were dried and dissolved in 50/50 (v/v) acetone/water for MS analysis. A low-pressure mercury lamp with an emission at 254 nm was placed 1.0 cm away from the nanoESI emitter (pulled from borosilicate glass capillaries) to initiate the Paternò-Büchi reaction. In order to differentiate C=C lipid isomers and to quantitatively determine their relative abundances, tandem mass spectrometry was applied. All MS experiments were performed on a 4000 QTRAP triple quadrupole/linear ion trap hybrid mass spectrometer (Sciex, Toronto, ON, CA).

### Statistical Analysis

All statistical analysis was conducted using the Microsoft Excel software (2010). Graphs represent the mean and respective standard error of mouse groups. Each group consisted of five samples. The differences between experimental groups were assessed by one-way analysis of variance followed by the paired two-tailed Student's *t*-test.

## Results and Discussion

### Profiling of Fatty Acids in SC1 and SC2 Spinal Cords of Normal and EAE Mice

**Figure 1:**
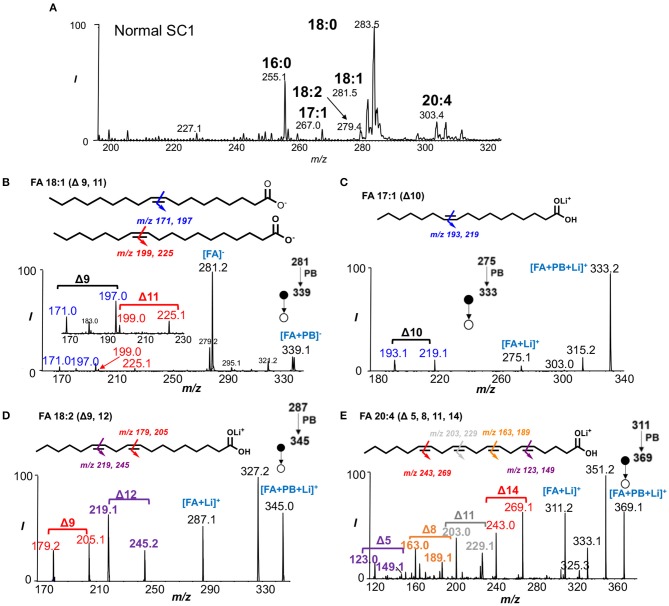
Characterization of fatty acids from crude lipid extract. **(A)** Rapid profiling of fatty acids in SC1 segment of normal mouse spinal cord. **(B)** PB-MS/MS of FA 18:1 shows two C=C location isomers (Δ9 and Δ11). **(C)** Structural identification of FA 17:1 using PB-MS/MS reveals the presence of C=C at Δ10. **(D)** PB-MS/MS of FA 18:2 unravels Δ9 and Δ12 C=C positions. **(E)** PB-MS/MS of FA 20:4 reveals the locations of four double bonds at Δ5, Δ8, Δ11, Δ13.

To perform rapid profiling of FAs, the solvent system of acetone/water was used since acetone acted as the PB reaction reagent for subsequent analysis. FAs were detected as deprotonated ions ([M-H]^−^) by nanoESI-MS in negative ion mode, including FAs 16:0 (*m/z* 255), 18:0 (*m/z* 283), 18:1 (*m/z* 281), 18:2 (*m/z* 279), and 20:4 (*m/z* 303) (Figure 1A, Supplementary Figures 1, 2). Saturated FAs 16:0 and 18:0 appeared to be the most abundant lipid species whereas FA 18:1 was the most abundant unsaturated FA in all spinal cord segments. Interestingly, the presence of FA 17:1 (*m/z* 267) was only noted in the SC1 segment of both normal and EAE mouse spinal cord tissues. FA 17:1 was found to be a trace component of the fat and milkfat of ruminants (Cooke et al., 1957) and would not naturally occur in animal or vegetable fat at relatively high concentrations (Beare-Rogers et al., 2001).

PB-MS/MS via collision-induced dissociation (CID) is capable for localizing C=C bond(s) position in unsaturated lipids without prior separations. In this method, a carbonyl group of choice such as acetone is first activated under UV irradiation at 254 nm wavelength. Once activated, the carbonyl group reacts with C=C in the unsaturated lipids to form an oxetane structure that subsequently gets ruptured by low-energy CID. This tandem mass spectrometric method releases a pair of characteristic diagnostic ions with a 26 Da mass separation that offer double bond position information since the two diagnostic ions in each pair bear the structures of an aldehyde and isopropene moiety at the original C=C location. Due to the mass difference between “O” and “C_3_H_6_,” the two fragment ions would always be separated by 26 Da when acetone is used as the reagent of choice in an MS/MS spectrum (Ma and Xia, 2014; Ma et al., 2016a). In this application, we directly subjected the crude spinal cord lipid extract for MS analysis in a shotgun lipid analysis approach to identify unsaturated FAs in the SC1 and SC2 spinal cords of normal and EAE mice. Consistent with previous reports (Ma et al., 2016a), FA 18:1 was found to consist of Δ9 (*m/z* 171.0, 197.0) and Δ11 (*m/z* 199.0, 225.1) isomers from detecting corresponding C=C diagnostic ions (Figure 1B). However, the application of PB-MS/MS in negative ion mode for FAs 17:1, 18:2, and 20:4 was not sensitive for detecting C=C diagnostic ions. Therefore, we applied PB-MS/MS for lithiated adduct ([M+Li]^+^) of these three FAs in the positive ion mode. Due to detection of a pair of C=C diagnostic ions at *m/z* 193.1 and 219.1, FA 17:1 has a C=C at Δ10 (Figure 1C); no C=C location isomer of this FA was found. Note that our method cannot differentiate straight fatty acyl chain from methyl branched structures (iso-FA 17:1). Therefore, it is possible that FA 17:1 is branched but this would not affect C=C location determination. Using the same approach, the C=Cs of FA 18:2 were determined at Δ9 (*m/z* 179.2, 205.1) and Δ12 (*m/z* 219.1, 245.2) (Figure 1D), while FA 20:4 was determined to have four double bonds located at Δ5 (*m/z* 123.0, 149.1), Δ8 (*m/z* 163.0, 189.1), Δ11 (*m/z* 203.0, 229.1), Δ14 (*m/z* 243.0, 269.1) (Figure 1E).

### Quantitative Analysis of Unsaturated Fatty Acids

**Figure 2:**
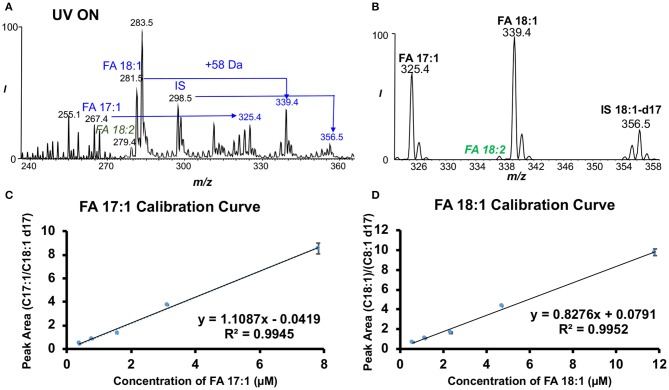
Approach for the absolute quantitation of unsaturated fatty acids in the mouse spinal cords. **(A)** Negative ion mode nanoESI mass spectrum of FAs that were photochemically tagged by acetone. **(B)** Fifty-eight dalton neutral loss scan (NLS) after acetone tagging. Calibration curves for each data point with three replicates of **(C)** FA 17:1 and **(D)** FA 18:1 based on NLS with FA 18:1-d17 as IS.

FA profiles showed notable differences among normal, EAE SC1 and SC2 spinal cord segments (Supplementary Figure 1). For unsaturated FAs, we employed an MS/MS transition, neutral loss scan (NLS) of 58 Da of the PB products to achieve fast quantitation (Ma et al., 2016b). As mentioned previously about the PB reaction, the reaction reagent of our choice, acetone (58 Da), was used to derive specific C=C information in unsaturated lipids. When coupled to MS/MS via CID, the loss of the 58 Da (tagged acetone molecule) can be observed as a major fragment upon CID of deprotonated ions of the PB products besides the C=C diagnostic ions (i.e., fragment ions at *m/z* 281.2 in Figure 1B). Due to this facile neutral loss of acetone in negative ion mode CID, the relative ion abundances of diagnostic ions are suppressed. Alternatively, CID of lithium ion adduct of the PB products in positive ion mode can be performed to enhance C=C diagnostic ion detection for confident structural determination. The 58 Da NLS in negative ion mode, however, enables a rapid and sensitive quantitation of unsaturated FAs (Ma et al., 2016b). In these experiments, FA 18:1-d17 (2.8 μM) was added as an internal standard (IS). Figure 2A shows the MS^1^ spectrum after the PB reaction. Clearly, all unsaturated FAs, such as FAs 17:1, 18:1, 18:2, 20:4, and IS, are detected as deprotonated ions ([M-H]^−^) along with +58 Da mass shift peak. Figure 2B demonstrates the effectiveness of 58 Da NLS in only capturing unsaturated FAs, while saturated FAs are not present, greatly improving selectivity and sensitivity for quantitation. Using 58 Da NLS calibration curves for each unsaturated FA of interest were obtained. Good linearity was acquired for these FAs ranging from *R*^2^ = 0.9925–0.9952 (Figures 2C,D and Supplementary Figure 3). By performing quantitation of unsaturated FAs with 58 Da NLS, concentrations of both monounsaturated and polyunsaturated FAs were discerned and expressed as μmol/g as discussed below. For more information regarding the quantitative analysis of unsaturated fatty acids, please see Supplementary Information (Supplementary Figure 4).

### Comparison of Unsaturated and Saturated Fatty Acids in Mouse Spinal Cords

**Figure 3:**
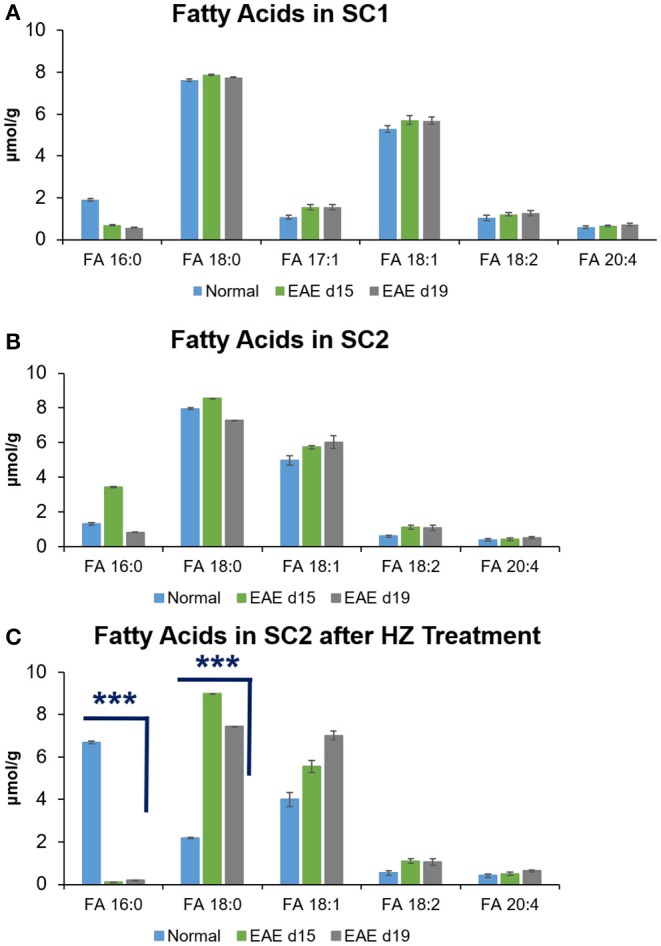
Quantitation of saturated and unsaturated FAs in mouse spinal cords of **(A)** SC1, **(B)** SC2, and **(C)** SC2 after HZ treatment. Error bars represent standard deviation, *n* = 5. Differences between the mouse spinal cords were confirmed via two-tailed Student's *t*-test (^***^p < 0.0005).

By using NLS 58 Da, quantitation of unsaturated FAs was performed (mean ± S.D., *N* = 5) for each spinal cord segment from controls and different disease stages. FA 17:1 was only observed in the SC1 portion of the spinal cord with concentration of 1.40 ± 0.11 μmol/g for all types of spinal cord tissues (Figure 3A). The concentration of FA 18:1 (5.56 ± 0.18 μmol/g) was much higher than that of FAs 18:2 (1.17 ± 0.11 μmol/g) and 20:4 (0.66 ± 0.05 μmol/g) in SC1 tissue segments (Figure 3A). The same trend was observed in the SC2 samples before and after hydralazine treatment (Figures 3B,C). Using two-tailed student's *t*-test, the concentration of FA 18:1 (7.00 ± 0.20 μmol/g) in hydralazine treated EAE d19 mouse spinal cord was found to have a significant increase (*p* < 0.0004, *N* = *5*) comparing to the normal treated tissues (4.00 ± 0.33 μmol/g). Overall, FA 18:1 exhibited higher level in normal and diseased samples (with and without hydralazine treatment), while the concentration level of FAs 18:1, 18:2, and 20:4 were relatively consistent in all samples even after treatment was applied.

Quantitation of saturated FAs was s performed for FAs 16:0 and 18:0 by MS^1^ in negative ion mode. For these two FAs, calibration curves were obtained using the IS of FA 17:0 (2.4 μM) since FA 17:0 was not detected in the spinal cord tissues. Good linearity of *R*^2^ = 0.9967 and 0.9976 was obtained for FA 16:0 and FA 18:0 calibration curves, respectively. In SC1 segment of all types of spinal cord samples, the concentration range of FA 16:0 was 1.06 ± 0.04 μmol/g whereas FA 18:0 concentration was 7.75 ± 0.04 μmol/g. In SC2 portion of the spinal cord, the concentration of FA 16:0 and FA 18:0 was 1.86 ± 0.04 μmol/g and 7.92 ± 0.04 μmol/g, respectively. Notably, the two saturated FAs in the SC2 segment showed significantly altered concentrations (*p* < 0.0001, *N* = 5) after hydralazine treatment. In EAE d15 spinal cord samples without hydralazine treatment, the concentration of FA 16:0 (3.44 ± 0.04 μmol/g) was higher than the spinal cord tissues of normal (1.32 ± 0.07 μmol/g) and EAE d19 (0.83 ± 0.02 μmol/g). With hydralazine treatment, the concentration of FA 16:0 significantly decreased to 0.10 ± 0.01 μmol/g compared to the normal SC2 tissues treated by hydralazine (EAE6.69 ± 0.07 μmol/g). In hydralazine treated EAE d19 samples, concentration of FA 16:0 also reduced significantly to 0.20 ± 0.005 μmol/g (*p* < 0.0001, *N* = 5) compared to the normal tissues treated by hydralazine. For FA 18:0, the concentration level (7.93 ± 0.04 μmol/g) of it varied less before hydralazine treatment for normal and diseased SC2 samples. However, after hydralazine treatment was applied, the concentration level of it increased significantly (*p* < 0.0001, *N* = 5) in the diseased samples of EAE d15 (8.98 ± 0.01 μmol/g) and EAE d19 (7.45 ± 0.005 μmol/g) compared to the normal treated by hydralazine (2.19 ± 0.03 μmol/g). Overall, the trend of change of FAs 16:0 and 18:0 was fairly similar in both spinal cord segments and changes in concentration level were evident after treatment. These differences insinuated that lipids such as FAs could potentially have an impact on the development and progression of multiple sclerosis. Furthermore, hydralazine could mediate the changes of FAs in the disease of multiple sclerosis as the levels of FAs varied after treatment.

### Determination of the Effects of Hydralazine Treatment on Fatty Acid Isomers in SC2

**Figure 4:**
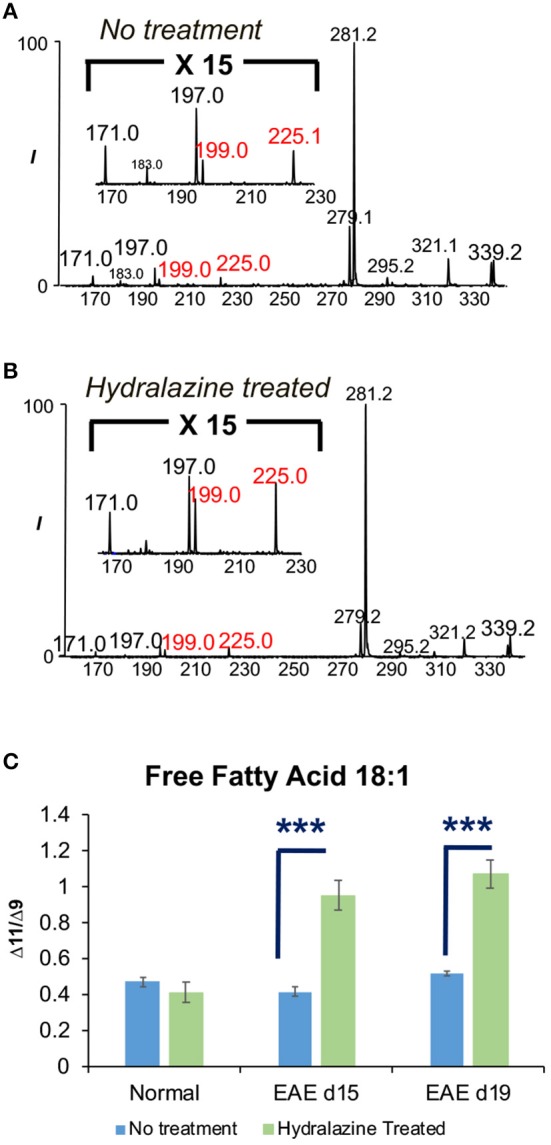
The effects of hydralazine treatment on the isomeric ratio of FA 18:1. **(A)** FA 18:1 in SC2 segment without hydralazine treatment. **(B)** FA 18:1 in SC2 segment with hydralazine treatment. **(C)** Comparison of Δ9 and Δ11 isomers of FA 18:1 in normal, EAE d15, and EAE d19 mouse spinal cords with/without treatment. Error bars represent standard deviation, *n* = 5. Differences between the mouse spinal cords were confirmed via two-tailed Student's *t*-test (^***^p < 0.0005).

FA 18:1 was found to contain two C=C location isomers, viz. Δ9 and Δ11 isomers, from all samples. The representative PB-MS/MS data for isomer identification can be found in Figure 1B for normal SC1 sample and Figures 4A,B for hydralazine treated and non-treated normal and EAE spinal cord tissues. Therefore, we further investigated changes in isomeric composition of FA 18:1 (Δ9 vs. Δ11 isomers) by performing relative quantitation of the two isomers. Relative isomer ratios were obtained by calculating the abundance ratio of the diagnostic ions of Δ9 and Δ11 isomers in FA 18:1 as in [Rel. C_Δ9_/C_Δ11_ = (I_171_+I_197_)/(I_199_+I_225_)]. There was no significant difference in the isomeric ratio of FA 18:1 in normal mouse SC2 spinal cord tissues without treatment and the normal ones treated with hydralazine. The isomeric ratio of Δ9 and Δ11 in FA 18:1 of treated SC2 spinal cord tissues differed significantly from the non-treated diseased samples. Significantly elevated concentrations of FA 18:1 (Δ11) were found in diseased spinal cord tissues after treated with hydralazine at d15 and d19 stages. These data suggested that the two C=C location isomers of FA 18:1 were altered in multiple sclerosis with hydralazine application (Figure 4C). This valuable piece of information would not be available without the capability of differentiating and quantifying C=C location isomers, which offers new insight into monitoring multiple sclerosis and treatment.

## Conclusion

Multiple sclerosis is a serious neurodegenerative disease that has treatments with only limited effects currently. A rising amount of evidence has indicated a link between the disease and lipids, suggesting that lipids may be a suitable diagnostic or therapeutic target in multiple sclerosis. Serving as the key building blocks of complex lipids, FAs are essential for maintaining the proper functions of all living organisms. There has been a lack of study on the possibility of FAs, especially their isomeric compositions, in playing a role in multiple sclerosis. With the novel methodology (the PB reaction coupled with tandem MS technique) that can identify and characterize C=C isomers, not only we have obtained the profiles of FAs in a shotgun lipid analysis manner, but also determined if C=C isomers play a role in the animal model of multiple sclerosis. Utilizing the 58 Da NLS, absolute quantitation of unsaturated FAs in the tissues was achieved, which provided insights into the levels of concentration changes in various tissues. We have successfully uncovered the various FA changes when comparing the normal and EAE spinal cord issues at different disease stages and the hydralazine treated samples. In addition, we have identified that isomer of FA 18:1 may potentially serve as a diagnostic marker for the disease. Therefore, the results from this study could potentially be extrapolated for biomarker application purposes as FAs and their isomeric composition could be monitored for multiple sclerosis progression as well as for the disease treatment effects. Finally, we have paved the way that offers an alternative methodology that investigates and quantifies lipids and their isomers in a potentially disabling disease that affects the central nervous system such as multiple sclerosis. Hence, our method can potentially align with preclinical models of clinical situation to evaluate the roles of lipids and their isomers in improving the understanding of the disease process and help define new safe and effective therapeutics.

## Data Availability Statement

All datasets generated for this study are included in the article/Supplementary Material.

## Ethics Statement

The animal study was reviewed and approved by IRB of Purdue University.

## Author Contributions

ZO and YX perceived the concept and planned the study. RS designed the animal model. LC executed the experimental analysis and analyzed the data. RT performed the animal study. LC, ZO, and YX wrote the manuscript.

### Conflict of Interest

The authors declare that the research was conducted in the absence of any commercial or financial relationships that could be construed as a potential conflict of interest.
