# Supplementary material for: Coupling the Paternò-Büchi (PB) Reaction With Mass Spectrometry to Study Unsaturated Fatty Acids in Mouse Model of Multiple Sclerosis
Source: Front Chem. 2019 Nov 26;7:807. doi: 10.3389/fchem.2019.00807 (PMC6901994; doi:10.3389/fchem.2019.00807)
Supplement: Supplementary file 1 [file Table_1.docx]

Supporting Information for

**Coupling** **the Paternò-Büchi (PB) Reaction with Mass Spectrometry to Study Unsaturated Fatty Acids in Mouse Model of Multiple Sclerosis**

Chong et al.

Includes: Supplementary Figures 1-4

Materials and Methods Extended


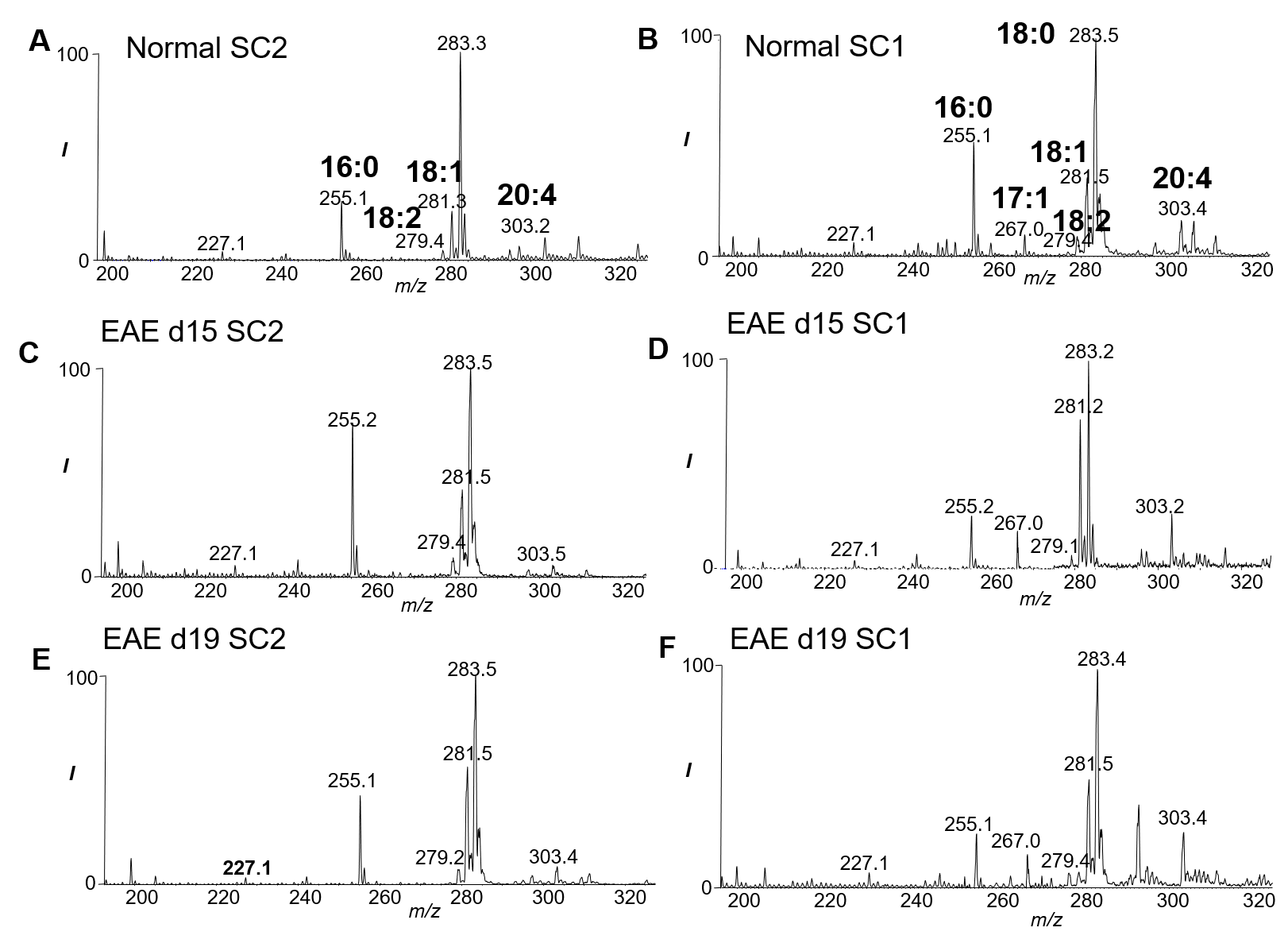


**Supplementary Figure 1.** Rapid profiling of fatty acids in (A) SC2 segment of normal mouse spinal cord, (B) SC1 segment of normal mouse spinal cord, (C) SC2 segment of EAE d15 mouse spinal cord, (D) SC1 segment of EAE d15 mouse spinal cord, (E) SC2 segment of EAE d19 mouse spinal cord, and (F) SC1 segment of EAE d19 mouse spinal cord for comparison. Saturated and unsaturated fatty acids with their corresponding *m/z* were observed in the mass spectrum.


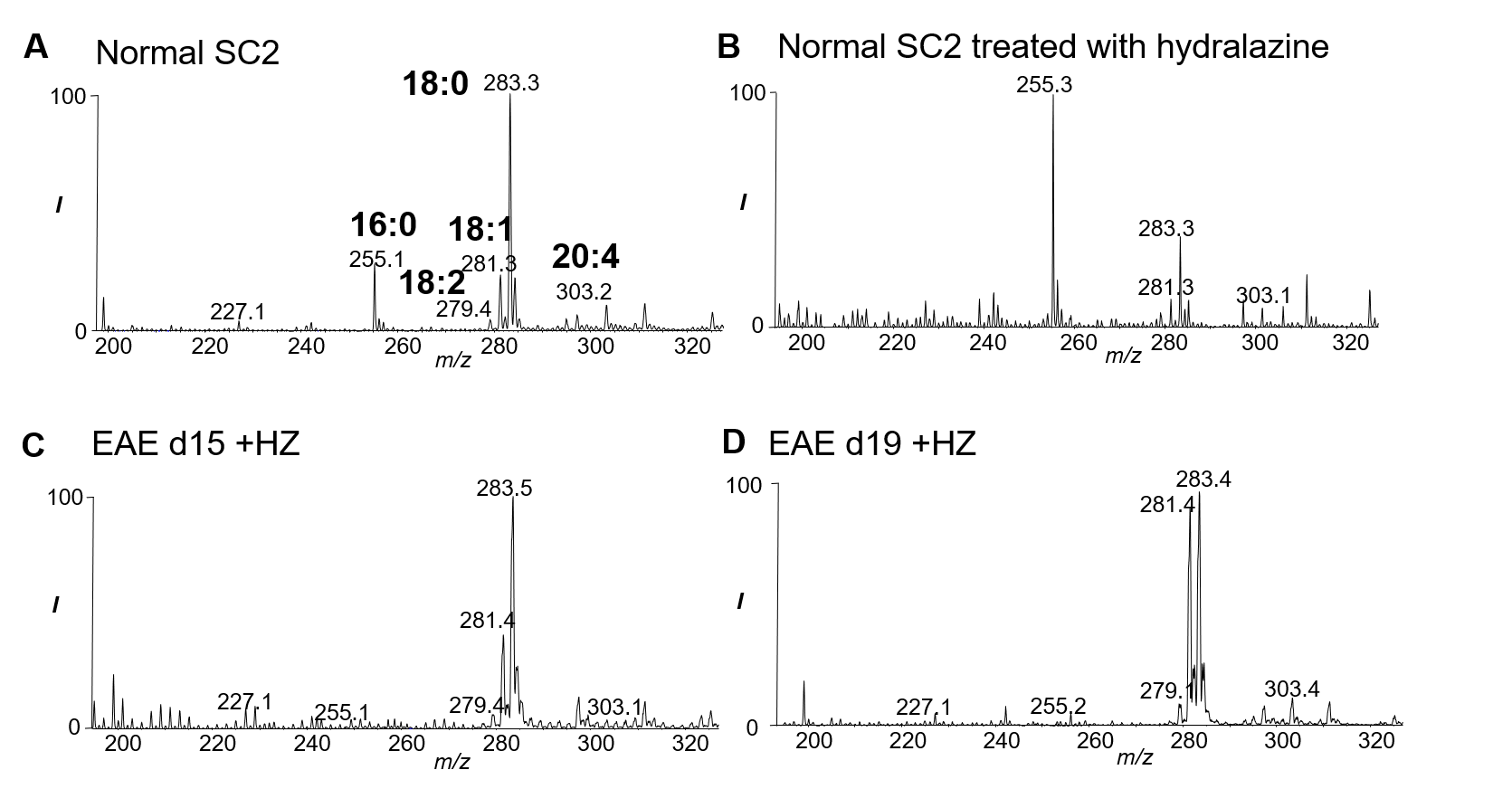


**Supplementary Figure 2.** Fatty acid profiles of A) SC2 segment of normal mouse spinal cord, B) SC2 segment of normal mouse spinal cord after treatment with hydralazine (HZ), C) SC2 segment of EAE d15 mouse spinal cord after treatment with hydralazine (HZ), and D) SC2 segment of EAE d19 mouse spinal cord after treatment with hydralazine (HZ) for comparison.


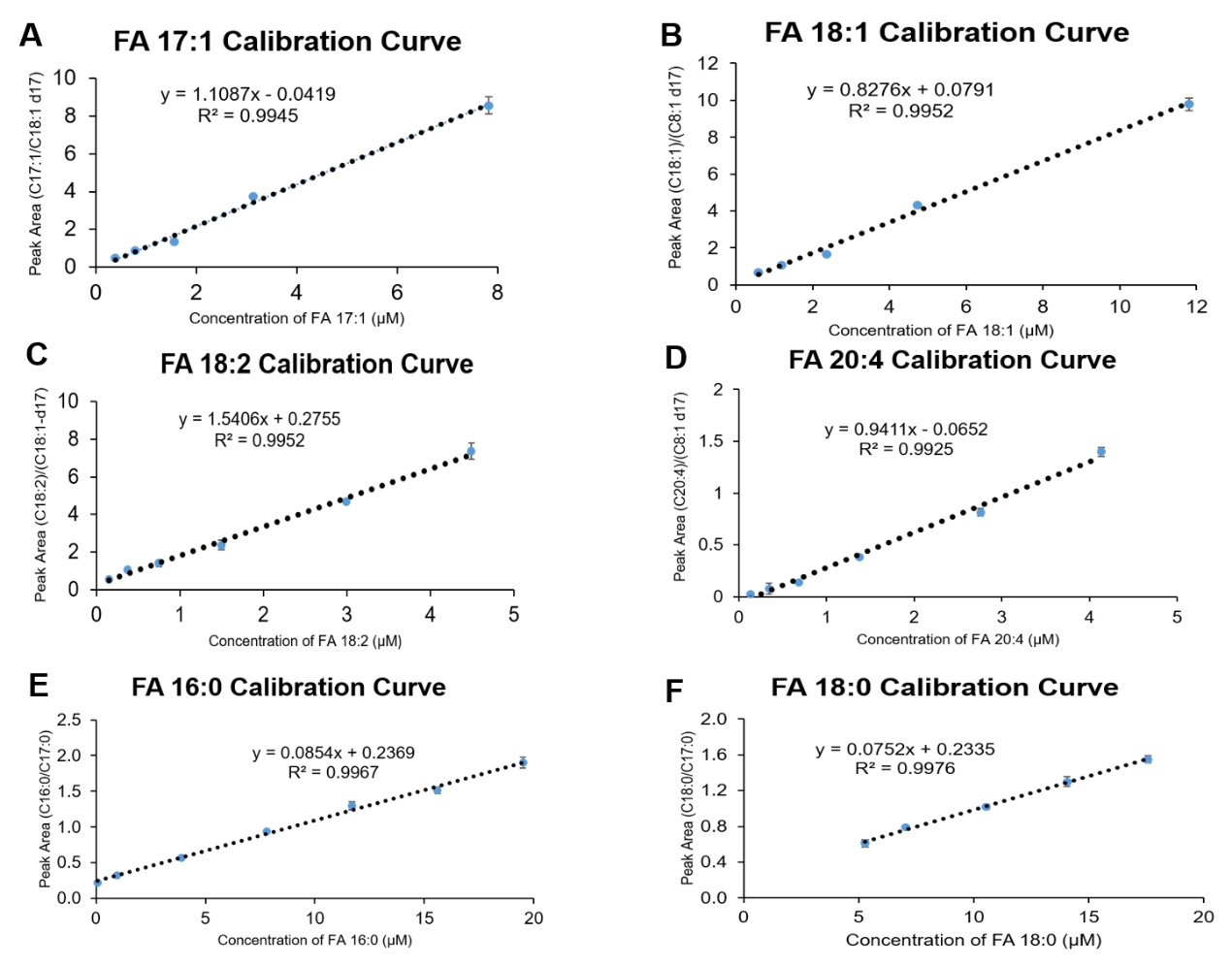


**Supplementary Figure 3.** Calibration curves generated for absolute quantitation of (A) FA 17:1, (B) FA 18:1, (C) 18:2, (D) FA 20:4, (E) FA 16:0, and (F) FA18:0.

**MATERIALS AND METHODS (Extended):**

**2.8. Fatty Acid Quantitative Analysis using PB-MS/MS**

For quantitative analysis of FAs, extracts were dried and dissolved in 50/50 (v/v) acetone/water. In order to effectively detect FA by negative ion mode nanoESI-MS, 0.5% (v/v) NH_4_OH (28%-30% as NH_3_) was also added into all FA solutions. Lipid solution was carefully pipetted into the back opening of the borosilicate glass tip (NanoESI tips) of ~10μm outer diameter. After the solution was loaded, a stainless-steel wire was placed into the tip to serve as the electric contact, with the nanoESI tip aligned with the MS sampling orifice. A low-pressure mercury lamp with an emission at 254 nm (Model No.: 80-1057-01, BHK, Inc., CA, USA) was placed 1.0 cm away from the nanoESI emitter to start the photochemical reaction process. All MS experiments were performed on a 4000 QTRAP triple quadrupole/linear ion trap (LIT) hybrid mass spectrometer (Sciex, Toronto, ON, CA). This instrument is capable of performing analysis in the triple quadrupole mode such as neutral loss scan (NLS) and LIT mode for MS/MS experiments. Typical instrument parameters were as follows: nanoESI voltage, -1200 to -1800V; curtain gas, 10 psi; interface heater temperature, 40°C; declustering potential: -30 V. For NLS, a CE of 35 V was utilized [1, 2]. For LIT MS/MS mode, precursor ion isolation width was set to 1.5 Th, and the precursor intensity was kept at around 4×10^6^ counts. The ion injection time was 10-200 ms. The collision energy (CE) used for PB reaction products of FAs was optimized to be 35 V (beam-type CID).

**2.9. Data Analysis**

Instrument control, data acquisition, and data processing were performed using the *Analyst* 1.6.2 software equipped on the QTRAP 4000 mass spectrometer. All data shown in this study were an average of 50 scans. Analyst allowed extraction of MS data with *m/z* values and corresponding peak areas from NLS, which were used for quantitative analysis. A step-by-step guide of how the data were obtained by the user from the *Analyst* software to perform quantitative analysis is illustrated below:

**Step 1:** Highlight the desired area in the chromatogram

**Step 2:** The user sees the desired peaks generated by the software

**Step 3:** The user clicks the “List Data” option from a drop-down menu and the software generates values for the peak area that the user can export for quantitative analysis


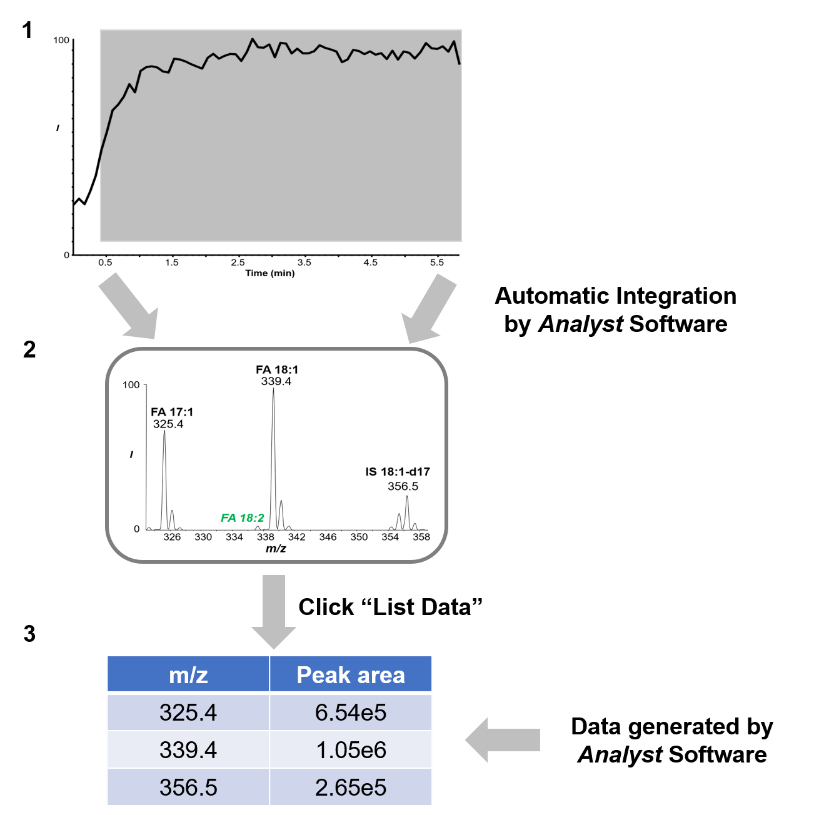


**Note:** a copy of the data integration and extraction performed by the user using the *Analyst* software is provided below:


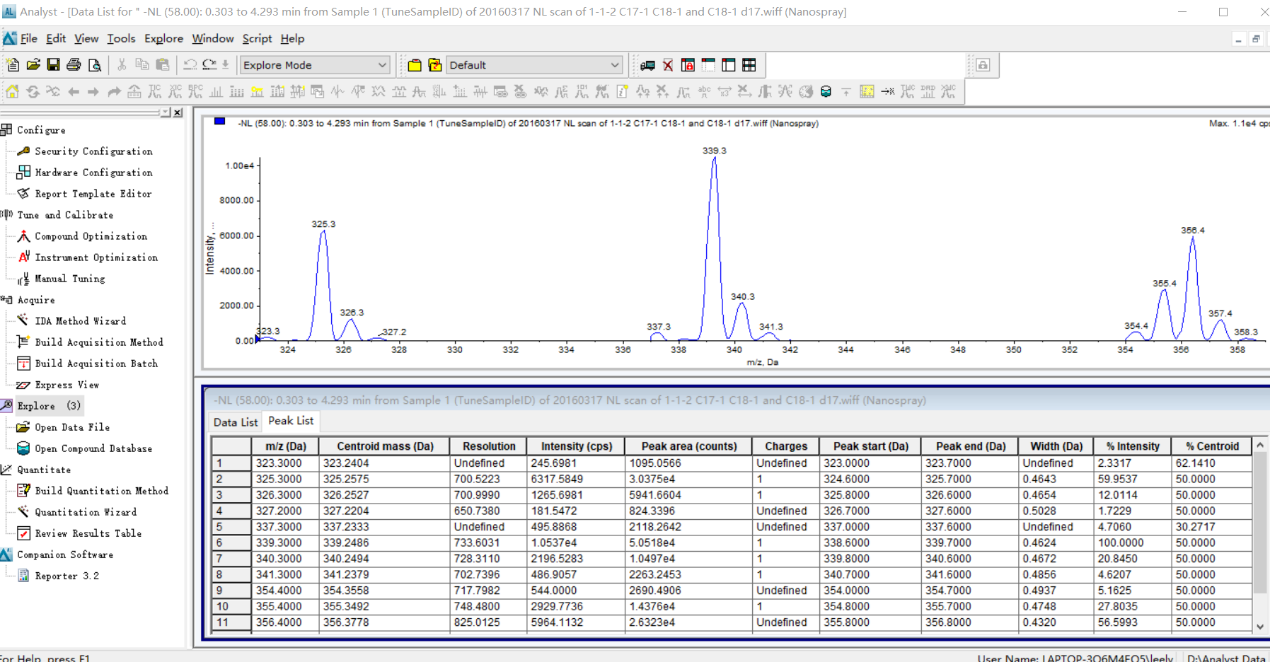


**Supplementary Figure 4.** Schematic of the quantitative analysis procedure using the *Analyst* software

**References:**

1. Ma, X. and Y. Xia, *Pinpointing double bonds in lipids by Paterno-Buchi reactions and mass spectrometry.* Angew Chem Int Ed Engl, 2014. **53**(10): p. 2592-6.

2. Ma, X., et al., *Photochemical Tagging for Quantitation of Unsaturated Fatty Acids by Mass Spectrometry.* Anal Chem, 2016. **88**(18): p. 8931-5.
